# Co-Designing a Volunteer-Delivered Behavioural Activation Intervention for Older Adults Living with Chronic Pain in Regional and Rural Australia: A Protocol for a Concept Mapping Study

**DOI:** 10.3390/nursrep16100349

**Published:** 2026-09-25

**Authors:** Laura Hynes, Sabine Pangerl, Phoebe George, Richard Gray, Lauren Blekkenhorst, Irene Ngune, Asan Akpan, Michael Hunter, Rosemary Saunders, Martin Jones

**Affiliations:** 1 School of Nursing and Midwifery, Edith Cowan University, Bunbury, WA 6230, Australia; s.pangerl@ecu.edu.au (S.P.); i.ngune@ecu.edu.au (I.N.); rosemary.saunders@ecu.edu.au (R.S.); martin.jones@ecu.edu.au (M.J.); 2 School of Population and Global Health, The University of Western Australia, Perth, WA 6009, Australia; phoebe.george@uwa.edu.au (P.G.); asangaedem.akpan@health.wa.gov.au (A.A.); 3 School of Nursing and Midwifery, La Trobe University, Melbourne, VIC 3086, Australia; r.gray@latrobe.edu.au; 4 School of Medical and Health Sciences, Edith Cowan University, Joondalup, WA 6027, Australia; l.blekkenhorst@ecu.edu.au; 5 Busselton Health Study Centre, Busselton Population Medical Research Institute, Busselton, WA 6280, Australia; michael.hunter@health.wa.gov.au

**Keywords:** chronic non-cancer pain, regional health, rural health, concept mapping, behavioural activation, psychosocial intervention, community-based care

## Abstract

**Background/Objectives:** Chronic non-cancer pain is a complex and highly prevalent condition that can cause significant physical, psychological, and social burdens on individuals and families, while placing increasing strain on health systems. This study aims to map and compare the perspectives of key stakeholders on the barriers, facilitators, and practical considerations relevant to the potential future development of a volunteer-delivered Behavioural Activation model of care for older adults living with chronic non-cancer pain in regional communities. **Methods:** Using Trochim’s structured concept mapping methodology, this mixed-methods, participatory study engages participants from three stakeholder groups: older adults with lived experience of chronic non-cancer pain, community (lay) volunteers and health professionals. Approximately 20–30 participants will be recruited, with around 7–10 participants from each stakeholder group, using community and professional networks, local referrals, and social media. Recruitment will remain flexible to support balanced representation across groups. Concept mapping will be used to compare perspectives on barriers, facilitators and implementation considerations relevant to a future volunteer-delivered model of care. The methodology comprises six stages: preparation, generation of statements, structuring of statements, representation, interpretation and utilisation. **Conclusions and Expected Results:** By exploring stakeholder perspectives, this study will identify priorities, barriers, facilitators and implementation considerations for community volunteer involvement in low-intensity psychosocial support. Findings may inform the development of future volunteer-delivered models and subsequent intervention and implementation studies, including structured training pathways and potential micro-credentialing for community volunteers in regional and rural settings. This study was retrospectively registered with the Open Science Framework Registries on 30 June 2026, following commencement of data collection for the brainstorming phase but prior to completion of the subsequent sorting, rating, representation and interpretation phases and prior to formal data analysis.

## 1. Introduction

### 1.1. The Problem

Chronic non-cancer pain (CNCP) is defined as pain that persists or recurs for more than three months, or beyond the normal healing time of an injury or illness in the absence of active cancer or cancer-related disease [1]. It is distinct from cancer-related pain or palliative/end-of-life pain, which have different treatment needs and care goals [1]. It is a multifactorial condition influenced by biological, psychological, and social factors and includes both chronic primary and chronic secondary pain conditions unrelated to malignancy [2]. Persistent pain can significantly restrict participation in both enjoyable and routine activities, ultimately diminishing overall wellbeing. Chronic pain encompasses heterogeneous conditions with differing symptom profiles and clinical needs [3]. In this study, these conditions are considered collectively because the focus is on shared barriers, facilitators and implementation considerations for behavioural and psychosocial support, rather than condition-specific treatment effects.

CNCP affects approximately one in four adults globally, with prevalence of 20% in the United States [4], up to 33.7% in Europe [5] and 18% in developing nations [6]. These prevalence estimates should be interpreted with caution, as they are derived from different populations, time periods and definitions of chronic pain. In Australia, the latest data shows that CNCP affects the quality of life of over 3.4 million people and imposes a considerable economic burden [7]. Approximately 3.4 million Australians were living with CNCP in 2020, and, without changes to current prevention and management strategies, this number is projected to increase to 5.2 million by 2050, with an estimated annual economic cost of $215.6 billion [8,9]. Notably, approximately one million older Australians were reported to be living with CNCP, with prevalence rates nearly double those observed in the working-age population. In Australia, older non-Indigenous Australians are typically defined as individuals aged 65 years and over [10]. In the South West region, the most recent available estimates indicate that musculoskeletal conditions accounted for 16.6% of total disease burden, with an age-standardised disability-adjusted life year rate of 32.5 per 1000 population [11]. Chronic pain prevalence has not been directly measured in the South West region; therefore, available musculoskeletal burden data provides only an indirect indication of the regional impact of chronic pain. The burden of CNCP is expected to increase further, largely attributed to factors such as an aging population, increased rates of multimorbidity and improved survival rates following health conditions that often lead to CNCP [12].

International guidance for chronic pain supports an individualised, multidisciplinary approach that may include education, physical activity and psychological therapies [3]. For CNCP, the National Institute for Health and Care Excellence (NICE) recommends considering Acceptance and Commitment Therapy (ACT) or Cognitive Behavioural Therapy (CBT) delivered by appropriately trained healthcare professionals [13,14]. However, accessing specialist health workers in regional and rural communities is difficult, as they tend to live and work in metropolitan communities. In Australia, prescribed opioid use is higher in rural and regional areas than in metropolitan areas. Medicare (Australia’s public health insurance scheme) prescription data show that of the relevant general practice consultations, rural areas had the highest rates of prescribed opioid use (72%), followed by regional areas (68%), while metropolitan areas had the lowest rates (65%) [15]. Emerging evidence suggests that this disparity is influenced by healthcare system factors and access to services close to home rather than clinician knowledge alone [9]. Thomas & Benson [16] identified a range of structural barriers to evidence-based pain management in rural settings, including time constraints, Medicare remuneration models, limited access to multidisciplinary pain services, and reduced availability of non-pharmacological treatment, specialist consultation, and referral pathways.

### 1.2. The Solution

This challenge highlights the need to explore alternative treatment options for pain management. Richards & Rhodes [17] aimed to establish the efficacy and cost-effectiveness of a much simpler approach than CBT, Behavioural Activation (BA). BA is a structured psychological intervention that commonly incorporates mood monitoring and activity scheduling to support engagement in meaningful and reinforcing activities [18]. Evidence for BA in chronic pain remains limited. A scoping review conducted by members of our research team identified 15 studies, including three randomised controlled trials, examining BA in relation to chronic pain [19]. While several studies reported improvements in pain-related outcomes, the evidence was heterogeneous in study design, intervention content and delivery, and the randomised trials were judged to be at high risk of bias. Adverse events were not reported, limiting conclusions regarding effectiveness and safety. More recent trials of BA or BA-informed approaches in chronic pain populations have since emerged [20,21,22]; however, heterogeneity in populations, intervention content, providers, delivery formats and comparators means the certainty of effects remains unclear [23]. Consequently, progression directly to a definitive effectiveness trial of volunteer-delivered BA for older adults with chronic pain would be premature. Before feasibility testing can be undertaken, greater clarity is needed regarding the proposed delivery model and its implementation context, including anticipated barriers and facilitators, workforce requirements, role boundaries, supervision, safety processes and support structures. The present Group Concept Mapping study therefore represents a preparatory step in intervention development, bringing together stakeholder perspectives to identify these implementation considerations and inform the design of a subsequent feasibility study.

Preliminary engagement with the Busselton Population Medical Research Institute (BPMRI) Community Advisory Committee and the broader South West community identified limited access to non-pharmacological pain support, highlighting the need for more accessible, community-based options. Community members particularly valued non-hierarchical, peer-level support that provided greater opportunities to discuss their experiences than time-limited healthcare consultations. These discussions identified trained community volunteers and peers as a potentially acceptable workforce for contributing to the delivery of BA within an appropriately supported model of care. This proposition is informed by previous Australian research on co-designing peer-delivered BA in farming communities, which demonstrated that trained non-clinical community members could deliver a structured BA intervention within defined governance, supervision and safety arrangements [24]. Although this work was undertaken in the context of depression and low mood rather than chronic pain, it provides a useful precedent for community-based task-sharing while reinforcing the need to establish population and context-specific implementation requirements before such a model is tested in older adults with chronic pain.

## 2. Materials and Methods

### 2.1. Aim

The aim of this study is to map and compare the perspectives of key stakeholders on the barriers, facilitators, and practical considerations for developing a volunteer-delivered BA model of care for older adults living with CNCP in regional and rural communities. In this study, co-design refers to stakeholder participation in generating, prioritising, and interpreting these perspectives to identify the principles and practical considerations that should inform subsequent development of the model; detailed intervention development, operationalisation, and feasibility testing are outside the scope of the present protocol and study.

### 2.2. Why Concept Mapping?

For this study, two mixed-methods approaches were considered: Concept mapping [25] and the Delphi technique [26]. While both methods are designed to capture stakeholder perceptions regarding a particular issue, they differ in purpose and outcome. The Delphi technique is primarily used to gather expert opinion and facilitate consensus [26]. Although useful for establishing agreement, it is less suited to exploring differences in perspectives across stakeholder groups. In contrast, concept mapping enables the generation, organisation, and comparison of ideas from diverse stakeholders, while simultaneously identifying areas of consensus and divergence [25]. Accordingly, concept mapping was selected as the most appropriate methodology to address the study aim. The protocol was developed with consideration of the emerging ConMapT reporting guidance for Trochim’s concept mapping methodology [27].

Concept mapping is widely recognised within health services research and implementation science as a rigorous participatory mixed-methods approach for examining complex phenomena and identifying priorities for intervention, policy, and practice [28,29,30]. As a self-contained mixed-methods methodology, it integrates qualitative and quantitative data collection and analysis to generate conceptual representations of stakeholder perspectives [31]. This makes it particularly well suited to identifying the barriers and facilitators associated with implementing behavioural activation across regional and rural community networks. Furthermore, concept mapping produces clear visual outputs that support stakeholder engagement, co-design, and decision-making, ensuring that the resulting model of care is evidence-informed, socially acceptable, and grounded in community priorities.

Integration of the mixed methods will occur throughout the concept mapping process. Qualitative ideas generated during brainstorming will be translated into quantitative sorting and rating data and analysed using multidimensional scaling and hierarchical cluster analysis. The resulting spatial and cluster structures will be interpreted alongside the original statement content and participant ratings. Areas of convergence and divergence will be identified and discussed, with pattern-match analyses used to examine differences in importance ratings between stakeholder groups.

### 2.3. Study Setting and Population

The study will be conducted in regional and rural areas of Western Australia in collaboration with the Busselton Population Medical Research Institute (BPMRI). Recruitment will focus on two locations: the City of Bunbury, classified as a Modified Monash Model (MMM) 2 area, and the City of Busselton, classified as an MMM3 area. According to the Australian Government Department of Health Disability and Ageing [32] MMM2 areas comprise regional centres that are within or adjacent to a town with a population of more than 50,000 residents, whereas MMM3 areas are regional towns that are not in close proximity to a major city and have populations between 15,000 and 50,000 residents.

Recruitment, data collection, and stakeholder engagement will be undertaken at the Edith Cowan University Busselton Campus and through secure online platforms, including videoconferencing and web-based data collection tools. The study involves three stakeholder groups: (1) health professionals, (2) community (lay worker) volunteers, and (3) older adults aged 65 years and over living with CNCP.

#### 2.3.1. Professional Group

Health professionals in the region are eligible to participate if they are currently practising or have recent experience (within the past two years) in healthcare delivery or research related to CNCP, mental health, or aged care. Eligible professions include nurses, nurse practitioners and allied health care professionals (exercise physiologists, physiotherapists, pain specialists, geriatricians, psychologists, dietitians, occupational therapists, psychiatrists, social workers, pharmacists, osteopaths and general practitioners). Recruitment will seek a breadth of professional perspectives to capture potential enablers and barriers to volunteer-delivered Behavioural Activation from a health system and service perspective. Professional differences will only be explored descriptively where relevant; discipline-specific comparisons are not a primary aim of the study.

#### 2.3.2. Volunteers Group

Community (lay worker) volunteers are unpaid members of the community who, despite lacking formal healthcare qualifications, receive role-specific training to provide practical, emotional, social, or health-related support within their communities. For this study, community (lay worker) volunteers will be recruited from regional and rural areas and included members of the BPMRI volunteer and consumer groups, together with participants from the Busselton Healthy Ageing Study (BHAS) who did not report chronic pain [33]. These individuals formed the initial community consultation group, building on a series of community engagement activities undertaken to ensure that the proposed research and methodological approach aligned with community priorities and needs. Additionally, recruitment occurs through local community networks and word of mouth to broaden participation beyond research-engaged cohorts; however, some selection bias may still remain, as participants may be more motivated, health-literate, or engaged with community health initiatives than the wider population.

The inclusion of community (lay worker) volunteers reflects the established partnership with BPMRI and recognises the important contribution that community members can make to the co-design of sustainable models of care. Their involvement ensures that the study is informed not only by the perspectives of older adults living with CNCP but also by community members who may ultimately contribute to the delivery of the intervention. This broader stakeholder representation strengthens the co-design process and supports the development of a volunteer-delivered Behavioural Activation model of care that is acceptable and responsive to the needs of regional and rural communities.

#### 2.3.3. Lived-Experience Group

Eligible older adults aged ≥65 years with lived experience of CNCP are recruited through the BHAS cohort. The rationale for including participants aged 65 years and older is supported by data from the Australian Government Department of Health and Aged Care. This evidence highlights that chronic pain is highly prevalent in this age group, affecting approximately one in three older Australians [7]. Participants will be required to report pain that has persisted or recurred for more than three months, consistent with the Australian Institute of Health and Welfare definition of CNCP outlined in the introduction [1]. This study focuses on older adults experiencing chronic non-malignant pain, with particular emphasis on musculoskeletal pain, arthritis, and other persistent pain conditions lasting longer than three months.

Inclusion criteria:

- Older adults aged ≥65 years;
- Musculoskeletal pain (e.g., low back pain, neck pain, osteoarthritis, joint pain);
- Fibromyalgia and widespread pain syndromes;
- Chronic headaches or migraines;
- Neuropathic pain (e.g., diabetic neuropathy, post-herpetic neuralgia);
- The cognitive capacity to provide informed consent independently.

Exclusion criteria:

- Certain pain conditions were excluded due to their distinct clinical trajectories or mismatched care requirements:
- Cancer-related pain, which typically necessitates complex multidisciplinary and/or palliative care.
- Acute pain (<3 months’ duration), such as post-surgical or post-trauma pain, which usually resolves with standard medical treatment.

Researchers will use a screening tool to confirm eligibility against the inclusion and exclusion criteria at each phase of the research if additional recruitment is required. Capacity to provide informed consent independently is assessed by the research team during the screening process, based on participants’ ability to understand the study information, explain the nature of participation, and communicate a voluntary decision to participate. No formal cognitive assessment tool will be administered. Where sensory, digital-access, or other participation limitations are identified, reasonable accommodations will be provided, including step-by-step written and video guides, telephone and in-person support, and access to institutional facilities where internet access or suitable devices are unavailable.

#### 2.3.4. Sample Size

Participants recruited for the brainstorming phase included older adults aged 65 years and over with lived experience of CNCP (n = 11), community (lay worker) volunteers (n = 7), and health professionals (n = 7), giving a total brainstorming sample of 25 participants. The planned sample size was informed by methodological recommendations for structured concept mapping and participatory mixed-methods research, which emphasise obtaining a breadth of stakeholder perspectives rather than achieving statistical power [34,35]. There is no universally accepted sample size for Group Concept Mapping; however, Trochim reported that studies commonly involved approximately 10–20 participants overall, while emphasising the importance of obtaining a breadth of perspectives relevant to the conceptual domain [25]. Given the inclusion of three distinct stakeholder groups, the study aimed to recruit approximately 7–12 participants from each stakeholder group. Participants who contributed to brainstorming are invited to participate in the subsequent sorting, rating, and interpretation phases; however, participation across phases is not assumed to be identical. Recruitment therefore remains open for subsequent phases, and additional participants may be recruited where required to maintain stakeholder representation or address attrition. Participation will be recorded separately for each study phase and stakeholder group. At the time of manuscript preparation, the sorting, rating, and interpretation phases had not yet commenced. Final participant numbers for each phase will be reported separately, including any attrition or variation in participation across phases.

Between-group comparisons will be exploratory and interpreted descriptively, particularly where group sizes are small or unequal. Incomplete sorting or rating data will be reviewed for usability and excluded from the relevant analysis where insufficiently completed.

### 2.4. Recruitment

#### 2.4.1. Professional Group

Recruitment will occur through multiple pathways, including professional networks, events, professional and social media platforms (e.g., Twitter, LinkedIn, Facebook) supported by snowball sampling to extend reach. This approach maximises diversity in representation. Individuals who expressed interest in the brainstorming phase were contacted directly by the research team, provided with written study information, and asked to return a signed consent form through returning a copy to the project email account or using a hard copy for those that have difficulty accessing or using technology. This recruitment approach will also be used for subsequent phases where additional recruitment is required because of attrition or under-representation. Any new participants recruited for subsequent phases will be provided with the relevant study information and informed consent will be sought prior to their participation.

#### 2.4.2. Volunteers Group

Community volunteers already engaged with the BHAS cohort were invited to participate in this study, under the approval and oversight of the BPMRI team. Invitations were distributed through BPMRI’s existing communication channels. Participants registered their interest in taking part via a Qualtrics link with the study lead directly contacting interested registrants and providing detailed study information. Informed consent was obtained electronically or in hard copy. Community volunteers were not asked to disclose any health information, as their role is to contribute perspectives on the importance, appropriateness, and social acceptability of a volunteer-led BA model of care. Where additional community volunteers are recruited for subsequent phases because of attrition or under-representation, the same recruitment process will be used and informed consent will be sought prior to participation.

#### 2.4.3. Lived-Experience Group

Participants were identified through the BHAS cohort database, facilitated by the BPMRI with ethical clearances obtained. A poster was distributed via BPMRI’s established digital communication channels. Participants registered their interest in taking part via a Qualtrics link. Interested individuals were contacted directly by members of the research team and provided with a detailed participant information sheet. Eligibility was assessed against the study inclusion and exclusion criteria.

As the study aims to capture the perspectives of individuals living with a range of CNCP conditions, recruitment was managed to ensure diversity within the participant group. Specifically, efforts were made to avoid the focus group being comprised predominantly of individuals with the same pain condition. This approach facilitated the inclusion of a broader range of experiences, challenges, and coping strategies, thereby strengthening the co-design process and ensuring that the volunteer-delivered behavioural activation model reflects diverse pain experiences and trajectories. Informed consent was obtained electronically or in hard copy. Where additional participants with lived experience are recruited for subsequent phases because of attrition or under-representation, the same recruitment and eligibility-screening process will be used, and informed consent will be sought prior to participation.

### 2.5. Scheduling Times for Concept Mapping Task

Members of the research team will coordinate with participants to arrange mutually convenient dates and times for each concept mapping activity. The brainstorming phase has been completed, while the prioritising, clustering, and interpretation phases are yet to occur.

### 2.6. Demographic Data

Demographic details from all participants include age, gender, location of work or residence, remoteness classification, highest level of education, Aboriginal and/or Torres Strait Islander status, and whether English was the main language spoken at home. Additionally, we will record the following details specific to each stakeholder group:

- Professional group—Primary professional role, healthcare setting, years of professional experience and management of chronic pain, specific training in chronic pain management, multidisciplinary work, proportion of chronic pain patients, types of chronic pain management
- Volunteers group—Living situation, relationship status, work status, health-related training, participation in community or volunteer programs, previous support of individuals with chronic pain
- Lived-experience group—Living situation, relationship status, work status, main income source, timeframe of chronic pain, formal diagnosis of chronic pain, types of chronic pain, long-term health conditions, use/access of pain services

### 2.7. Ethics Considerations

The study is conducted in accordance with the National Statement on Ethical Conduct in Human Research [36]. Compliance with these standards provides assurance that the rights, safety and wellbeing of research participants are respected. Ethics approval was attained from the University Human Research Ethics Committee on 27 November 2025 Research Ethics Management System Approval Number: 2025-06850-HYNES and Busselton Population Medical Research Institute Ethics Approval Number: BSN 25/14). Ethical considerations include informed consent which will be obtained electronically and via hardcopy for each phase, as well as the participants’ confidentiality and management of data. Informed consent has been obtained from participants enrolled to date and will be obtained from any additional participants prior to participation. Recruitment and data collection remain ongoing for the subsequent sorting, rating, representation, and interpretation phases. Focus groups were conducted in person and audio-recorded with participants’ consent. Participants were informed that confidentiality within focus-group discussions could not be guaranteed and were asked to respect the privacy of other participants by not disclosing information discussed during the session outside the group. Participant confidentiality will be strictly maintained throughout all stages of the research. Research data will be de-identified prior to analysis and reporting, and no individual participant will be identifiable in any publications, presentations, or reports arising from this study. All research data including transcripts, coded data, audio recordings, and exported Group Concept Mapping outputs are securely stored on a password-protected university server.

### 2.8. Risks Associated with Taking Part in the Study

Participants living with CNCP (lived-experience group) may experience psychological distress throughout the data collection. Discussing pain experiences, previous negative interactions with health services, or the emotional and social impacts of CNCP may evoke feelings of sadness, frustration, or vulnerability. To minimise these risks, discussions were facilitated with sensitivity, and participants were reminded that participation is voluntary and that they may pause, decline to answer questions, or withdraw at any time without consequence. A distress protocol was in place for the brainstorming phase and will also be applied during the interpretation phase. If significant distress occurs, AI Jones, a registered nurse with specialist mental health training and the designated observer, will provide immediate support. Serious psychological distress or suicide risk will be escalated to an appropriate health professional or emergency service where required. Participants will also be offered information about psychosocial support services available should they need them, including Healthdirect, Beyond Blue, and ReachOut, and encouraged to seek follow-up support from their General Practitioner or existing healthcare providers if appropriate. A follow-up check-in will be offered if indicated.

For professional participants, risks relate primarily to professional identity and reputational sensitivity rather than psychological harm. Sharing experiences related to service delivery challenges or resource constraints may raise concerns about how their views could be perceived by colleagues, employers, or the community. To mitigate these risks, all data will be de-identified, pseudonyms will be used in reporting, and no organisational names, locations, or identifying details will be linked to individual responses.

### 2.9. Potential Benefits of This Research

The primary beneficiaries of this initiative in the future are older adults living with CNCP pain in regional and rural Australia, who often encounter significant barriers to accessing support services due to geographic isolation and limited availability of trained healthcare professionals. Findings from the present study may inform the future development and feasibility testing of a volunteer-delivered BA model of care by identifying key implementation considerations, including barriers and facilitators, workforce requirements, role boundaries, supervision, safety processes and support structures.

### 2.10. Data Collection Procedure

The concept mapping process consists of six distinct phases. Participants will initially engage in a brainstorming session to generate ideas related to the study topic and subsequently complete online sorting and rating activities, including clustering and prioritisation of statements [34].

Phase 1: Preparation

The involvement of people with lived experience of CNCP, community volunteers, and health professionals provides a collaborative co-design approach, bringing together their perspectives to jointly identify and prioritise the barriers, facilitators, and practical considerations needed to inform the development of a future volunteer-delivered BA model of care. The initial step involved defining the focus prompt and identifying the participant groups. The focus of this study is “What would help or make it difficult for volunteers to deliver Behavioural Activation for older adults with CNCP in regional and rural communities?” In the preparation phase the three stakeholder groups representing different perspectives were determined: the professional group, the community volunteers group and the lived-experienced group.

Phase 2: Generation of Statements (Brainstorming)

Brainstorming activities were conducted between the 1st of March and 30th of April 2026 in focus groups lasting approximately 60–90 min. Separate focus groups were held for each of the three participant groups to ensure that perspectives from each stakeholder group were captured independently. A total of 6 focus groups were conducted, with multiple sessions held within some stakeholder groups to accommodate participant availability. Each focus group included a minimum of three participants, and stakeholder groups were facilitated separately to preserve group-specific perspectives. Sessions were facilitated by CI Hynes, AI Jones, and with AI George taking field notes to capture contextual observations and support interpretation of the brainstorming process. To minimise researcher influence, facilitators used the same predefined focus prompt and neutral probing, avoided leading participants, and encouraged all participants to contribute independently. This approach facilitated open discussion among participants with shared experiences and allowed for more nuanced identification of group-specific factors relevant to the research question. The guiding question was “What would help or make it difficult for volunteers to deliver Behavioural Activation for older adults with CNCP in regional and rural communities?”. Participants were invited to produce short, clear statements in response to this focus prompt. They were encouraged to avoid long or complex phrasing, conditional “if–then” formulations, make comparisons (e.g., “is similar to”), double negatives, or value-laden statements (e.g., “A useful approach would be…” [37]. Focus groups were audio-recorded to support the generation and extraction of participant-derived statements. Consistent with Group Concept Mapping methodology, full transcripts were not produced and no separate thematic analysis was undertaken, as the purpose of the brainstorming phase was to generate participant-derived statements for subsequent synthesis, sorting and rating [27]. Following the completion of the brainstorming sessions, one researcher independently listened to the audio recordings two to three times to identify and extract participant-generated statements verbatim. A second researcher reviewed the extracted statements against the audio recordings. Any discrepancies were discussed with the broader team and resolved through consensus. Consistent with structured concept mapping methodology, statements were required to represent a single, specific idea and be conceptually distinct from other statements to minimise overlap and redundancy [27]. The research team subsequently met to review the 101 generated statements. Statements were merged, removed, or reworded where they were duplicated, conceptually overlapping, unclear, or contained more than one idea, while retaining the original meaning and intent of participants’ contributions. An audit trail was maintained throughout the statement-synthesis process using spreadsheets and tracked-change documents to record decisions regarding statement retention, rewording, merging, and removal. The number of statements was also reduced to minimise participant burden and fatigue during the subsequent sorting and importance-rating activities. This process resulted in a final set of 60 statements.

Protocol registration occurred following completion of the brainstorming and statement-synthesis phases and prior to the subsequent sorting, rating, representation and interpretation phases. Accordingly, the statement-generation and synthesis procedures are reported retrospectively, while the remaining phases are described prospectively, as they have not yet occurred.

Phase 3: Structuring of Statements

In this phase structuring of statements involves two tasks: prioritisation and clustering. These tasks will be completed using the group concept mapping software Groupwisdom^TM^ (Build 2021.24.01), with all participants who participate in phase one and two receiving an individual emailed link providing direct access to the platform. The online component of the concept mapping study will be piloted with a small group (n = 5) to assess face validity, clarity, comprehensibility, and feasibility prior to broader distribution. The pilot group will include five individuals: one for whom English is a second language, one lay person with no health science background, one content/methodology expert, one healthcare professional, and one community member without a healthcare or research background. Pilot participants will not be included in the final study sample. The structuring phase involves compiling all statements from the three stakeholder groups and comprise two key individual participant-led activities:

- Prioritisation: Participants will rate statements for importance using a 5-point Likert scale (1 = least important; 5 = most important). Participants are advised not to consider the lowest category “unimportant” but “least important” to avoid a situation where participants refuse to place statements in the lowest category because they still consider them important [37].
- Clustering: Participants will group statements into clusters that make sense to them. Participants will sort the statements into clusters based on perceived similarity or conceptual relatedness. Cluster size will not be restricted; however, each statement may only be assigned to one cluster. Participants will be invited, but not required, to provide a label for each cluster that reflects the overarching concept represented by the grouped statements [25,37].

Phase 4: Representation

Individual sorting and importance ratings will be entered into the Group Concept Mapping software (GroupWisdom^TM^ Build 2021.24.01) in de-identified form and combined to generate a group-level output. Participant sorting data will first be transformed into a similarity matrix representing the frequency with which pairs of statements were sorted together. The similarity matrix will then be analysed using multidimensional scaling (MDS) to generate a two-dimensional point map, with statements that are more frequently sorted together positioned closer together. The stress value will be reported to indicate how well the two-dimensional configuration represents the original sorting data. Hierarchical cluster analysis will then be applied to the MDS solution to group statements into conceptually related clusters. A range of cluster solutions will be reviewed iteratively, considering the statements within each cluster, their conceptual coherence, and spatial relationships. Plausible solutions will be considered with stakeholders, and the final solution will be selected based on conceptual meaning, interpretability, and parsimony. Cluster labels and descriptions will then be refined collaboratively, with decisions documented to provide an audit trail. The final concept maps will display individual statements and their spatial relationships within clusters. Mean importance ratings and 95% confidence intervals will be calculated for individual statements and clusters, and statement and cluster bridging values will be examined to support interpretation of the conceptual structure.

Stakeholder group comparisons will be undertaken using mean importance ratings for individual statements and clusters. Pattern-match analyses will compare mean cluster importance ratings between stakeholder groups, with Pearson correlation coefficients used to indicate the similarity of rating patterns. Particular attention will be given to areas of convergence and divergence in perceived importance. If group sizes differ, analyses will be interpreted descriptively, with emphasis on the extent and pattern of differences rather than statistical significance.

Phase 5: Interpretation of Concept Maps

Once the number of clusters has been determined, preliminary materials for the interpretation sessions will be prepared. Up to eight candidate concept maps will be generated in the Group Concept Mapping software that need to be qualitatively interpreted and explained [25]. These candidate maps will represent alternative cluster solutions derived from the same underlying point map, rather than separate stakeholder-specific maps. These candidate solutions will be reviewed collaboratively by the research team and approximately 10–15 participants, representing a balanced mix of all stakeholder groups. During the interpretation session, participants will discuss the spatial organisation and thematic meaning of clusters, refine preliminary cluster labels, and consider the relative importance of clusters using the rating data [37]. Researchers and participants will work toward consensus on the concept map that best represents the key barriers, facilitators and practical implementation considerations for developing a volunteer-delivered BA model for older adults with CNCP in regional and rural communities. Insights generated during the interpretation sessions may inform further refinement of cluster labels and the generation of additional outputs to support interpretation of the findings [37]. The process used to determine the final concept map will be documented, including consideration of conceptual coherence, interpretability and stakeholder feedback.

Phase 6: Utilisation of Concept Maps

Findings from this concept mapping study will provide the stakeholder-informed basis for subsequent co-design activities to further develop the proposed volunteer-delivered BA model and its implementation requirements prior to potential feasibility testing. These subsequent co-design, development, and feasibility activities are outside the scope of the present study.

### 2.11. The Time Commitment Involved

Participant involvement in the study will typically take approximately 2.5 to 4 h. The brainstorming task takes between 60–90 min, while prioritisation and clustering will require approximately one hour. If participants also take part in interpreting of the concept maps, this will add an additional hour.

### 2.12. Study Status

Data collection prior to submission of this protocol occurred only for Phase 2 (Generation of Statements—Brainstorming). Phase 1 (Preparation) and Phase 2 have been completed; however, no formal data analysis had been undertaken at the time of submission. Phase 3 (Structuring of Statements—Sorting and Rating), Phase 4 (Representation), Phase 5 (Interpretation of Concept Maps), and Phase 6 (Utilisation of Concept Maps) had not yet commenced and remain prospective. Accordingly, data collection and participant recruitment for these phases had not been completed at the time of submission.

### 2.13. Limitations

This study is limited using convenience and snowball sampling, which may introduce sampling and selection bias. Participants within the target populations do not have an equal probability of being recruited, which may limit sample diversity and affect the transferability of findings. Individuals who are less willing to participate or who hold differing views may be underrepresented.

There is also a small possibility that some participants representing practitioner perspectives or policy and service delivery may have prior professional relationships with members of the research team. This could create a perception that participation is expected or obligatory. To minimise this risk, all potential participants were explicitly informed that involvement is entirely voluntary, that there are no professional or personal consequences for declining, and that they may withdraw at any time without consequences. This approach promotes transparency and reduces the potential for undue influence arising from pre-existing relationships.

A potential limitation of this mixed methods approach is that stakeholder interpretation may influence cluster labels and meanings beyond the statistical outputs, while the quantitative analyses may not fully capture nuanced qualitative perspectives. These limitations will be considered during interpretation and reporting.

Finally, prior engagement with the research team may have also introduced a risk of social desirability bias, which could influence participant responses. This is acknowledged as an inherent limitation of the study.

## 3. Conclusions and Expected Results

This study seeks to identify stakeholder perspectives, priorities, barriers, facilitators, and practical considerations relevant to the potential future development of a community-delivered behavioural activation model for older adults living with CNCP in regional and rural Australia. Using a participatory mixed methods approach, the study will generate a preliminary, stakeholder-informed conceptualisation of how the potential role of trained community volunteers could contribute to the delivery of low-intensity psychosocial support. The findings are intended to inform the design and refinement of future intervention and implementation research, including studies that may subsequently assess the feasibility, acceptability, effectiveness, and scalability of such a model. In the longer term, the findings may contribute to consideration of approaches to expanding access to evidence-based psychosocial support and informing workforce development initiatives, including structured training pathways and potential micro-credentialing for community volunteers.

## Data Availability

Data will be available on request from the authors due to privacy or ethical restrictions.

## Public Involvement Statement

Members of the BPMRI Community Advisory Committee contributed to the early development of the study by helping shape the research question and informing the selection of Group Concept Mapping to explore implementation needs. During the study, other community members and people with lived experience also contributed through the Group Concept Mapping process, providing stakeholder perspectives that informed the co-design activities undertaken within this study.

## Guidelines and Standards Statement

The protocol was developed with consideration of the emerging ConMapT reporting guidance for Trochim’s concept mapping methodology [27].

## Abbreviations

The following abbreviations are used in this manuscript:

CNCP	Chronic non-cancer pain
CBT	Cognitive behavioural therapy
BA	Behavioural activation
BPMRI	Busselton population medical research institute
MMM	Modified Monash model
BHAS	Busselton Healthy Ageing Study
